# Technological and Physical–Chemical Evaluation of Cotton Gauzes Impregnated with Semisolid Preparations for Wound Healing

**DOI:** 10.3390/biomedicines12040777

**Published:** 2024-04-01

**Authors:** Fabrizio Villapiano, Ritamaria Di Lorenzo, Rosa Sparaco, Elisa Magli, Francesco Frecentese, Sonia Laneri, Alessandra D’Orsi, Valeria Nele, Marco Biondi, Laura Mayol, Virginia Campani, Vincenzo Santagada, Giuseppe De Rosa

**Affiliations:** 1 Department of Pharmacy, University of Naples Federico II, Via Domenico Montesano 49, 80131 Naples, Italy; fabrizio.villapiano@unina.it (F.V.); rosa.sparaco@unina.it (R.S.); francesco.frecentese@unina.it (F.F.); ales.dorsi@studenti.unina.it (A.D.); valeria.nele@unina.it (V.N.); mabiondi@unina.it (M.B.); vincenzo.santagada@unina.it (V.S.); gderosa@unina.it (G.D.R.); 2 RD Cosmetics, Department of Pharmacy, University of Naples Federico II, Via Domenico Motesano 49, 80131 Naples, Italy; ritamaria.dilorenzo@unina.it (R.D.L.); slaneri@unina.it (S.L.); 3 Department of Public Health, University of Naples Federico II, Via Pansini 5, 80131 Naples, Italy; elisa.magli@unina.it; 4 Department of Advanced Biomedical Sciences, University of Naples Federico II, Via Pansini 5, 80131 Naples, Italy

**Keywords:** wound healing, wound dressing, rheology, corneometry, skin energy

## Abstract

Chronic wounds are marked by an extended healing period during which damaged tissues fail to undergo orderly and timely repair. Examples of chronic wounds encompass venous ulcers, pressure ulcers, and diabetic foot ulcers. The process of wound healing is complex and dynamic, relying on the interplay and response among various cells and mediators. In this study, four marketed wound dressing products based on cotton gauzes impregnated with different semisolid products (namely Betadine^®^ 10%, Connettivina^®^ Bio Plus Fitostimoline^®^ Plus, and Non-Ad^®^ gauzes) have been characterized for their physicochemical properties and ex vivo behaviors. More in detail, the pH and rheological features of semisolid formulations impregnating the gauzes were analyzed along with their ability to adhere to the gauzes. The most promising ones were selected and compared in ex vivo experiments on fresh pig skin. The pH measurements showed an acidic environment for all the tested solutions, albeit with variations in mean values, ranging from 2.66 to 4.50. The outcomes of rheological studies demonstrated that all the semisolid preparations impregnating the gauzes exhibited a pseudoplastic behavior, with significant differences in the pseudoplasticity index across the preparations, which is likely to influence their ability to adhere to the gauze. A rheological study in oscillatory mode revealed rheological behavior typical of a viscous solution only for the cream impregnating non-paraffin gauzes. The other products exhibited rheological behavior typical of a weak gel, which is expected to be advantageous as regards the capability of the semisolid preparation to create and maintain the space within the wound and to provide protection to the injured tissue. Results of ex vivo experiments demonstrated that Fitostimoline^®^ Plus was more effective than Connettivina^®^ Bio Plus in promoting both skin hydration and energy.

## 1. Introduction

Chronic wounds are characterized by a prolonged healing time in which the damaged tissues cannot be repaired in an orderly and timely manner. Examples of chronic wounds include venous ulcers, pressure ulcers, and diabetic foot ulcers [1].

Wound healing is a complex and dynamic process based on the reaction and interaction between cells and different mediators. The sequence of events occurring during the healing consists of hemostasis, inflammation, proliferation, and remodeling [2,3]. Different factors affecting physiological response and cellular function can interfere with these pathways at many levels. Local factors such as ischemic tissues, contamination, infection, but also age, nutrition, and pathology, including diabetes mellitus or metabolic diseases, are associated with delays in the wound healing mechanism [2,4]. Wound pH also plays an important role in the healing process and could be considered an indicator of the healing stage as well as the presence of potential infections. Researchers have reported that the pH of a chronic wound is in the range of 7.15–8.9 and topical preparations and dressings that acidify the wound bed increase the healing rate and reduce the risk of polymicrobial infections [5,6].

For wound care, several presidium dressings are available. These products are designed to be in contact with the wound, offering physical protection and preventing infections. Ideally, wound dressings should speed up the process of healing by promoting re-epithelialization and collagen synthesis as well as stimulating angiogenesis [7].

Currently, different types of wound dressings are clinically available to address these needs. The most common medical dressings are bandages and gauzes, made of woven or non-woven fibers of cotton, rayon, polyesters, and more recently knitted cotton fibers, too. Cotton dry gauzes act as a barrier against the external environment, protecting the wound from contamination and simultaneously absorbing the exudates from the wound bed. Since these dressings usually are not capable of water retention, their removal may be painful for the patient [2,3]. To address this issue, dressings have been impregnated with solution or semisolid preparations that recreate a proper moist environment on the wound bed. Thus, occlusive dressings promote a proper and effective healing process [8] due to the release of tissue growth factors that stimulate angiogenesis and prevent the high evaporation of the lesion site, thus reducing wound pain [9,10]. Furthermore, the addition of active ingredients for antimicrobial purposes allows for the prevention of bacterial infection. However, this traditional approach to wound care still presents some disadvantages, including the risk of tissue ischemia or necrosis and the need for frequent substitutions. Therefore, new technologies have been introduced. Advanced and smart dressings can deliver bioactive compounds to the wound site, speeding up the healing process. Also, smart materials have been studied, such as ROS-responsive materials, temperature-responsive materials, pH-responsive materials, or shape-memory materials, which can control different parameters related to wound healing [9].

Even though there is no univocal consensus on the ideal dressing characteristics, technical properties such as conforming to the wound surface, the filling of dressing cavities, and easy removal that avoids skin damage must be taken into account to recreate the optimal condition for tissue regeneration [11].

These features are deeply related to the rheological properties of the formulation, such as viscosity, elasticity, thixotropy, and flow [12]. Indeed, the rheological behavior affects the spreadability of semisolid products on the gauze, its adhesion to the skin, and the release profile of APIs into the skin [13,14].

In this study, we analyzed and compared different wound dressings consisting of impregnated gauzes (Table 1). We focused our attention on the following products: Betadine^®^ 10% gauze (Viatris Pharmaceuticals, Mérignac, France), Connettivina^®^ Bio Plus gauze (Fidia Farmaceutici S.p.A., Albano Terme, Italy), Fitostimoline^®^ Plus gauze (Farmaceutici Damor S.p.A., Napoli, Italy), and Non-Ad^®^ gauze (Eurospital S.p.A., Trieste, Italy). Betadine^®^ 10% gauzes are soaked in a 10% solution of povidone-iodine, a broad-spectrum biocidal agent widely used to prevent the microbial contamination of wounds and ulcers.

Actually, different povidone iodine-based formulations such as foam, ointment, liposomal hydrogel, dry powder spray, or solution are on the market to suit specific medical needs [15].

Connettivina^®^ Bio Plus gauze is a semisolid-impregnated gauze pad based on low-molecular-weight (200 kDa) hyaluronic acid (HA) and silver sulfadiazine 1%. HA is a major component of the extracellular matrix (ECM) and plays an important role in all phases of the wound-healing process. It keeps the wound moist, protecting it from dryness, and is a component of different preparations, including foams, gauzes, or creams. Silver sulfadiazine has antimicrobial properties; in particular, sulphadiazine is a bacteriostatic agent and silver nitrate acts on the endocellular structures. Therefore, the association is a useful tool against bacterial contamination. Both cream and cream-impregnated gauze pads containing hyaluronic acid and silver sulfadiazine 1% have proven to be effective in reducing wound size, protecting the surrounding skin, and preventing bacterial infection [16].

Fitostimoline^®^ Plus gauze is a medical dressing containing Rigenase^®^ associated with poly-hexanide (PHMB). Rigenase^®^ (Farmaceutici Damor S.p.A., Napoli, Italy) is an aqueous extract from *Triticum vulgare*, rich in oligosaccharidic components that have demonstrated anti-inflammatory and antioxidant activity, stimulate the formation of ECM components, and enhance keratinocyte activity, accelerating wound healing to stimulate the healing process in cutaneous diseases such decubitus ulcers, wounds, burns, and delayed scarring. Rigenase^®^ and polyhexanide-based dressings have been extensively employed. When applied as a cream or impregnated gauze, they create a barrier between the skin and the outside world, promoting quicker skin re-epithelialization and more efficient wound healing [11,17,18].

Non-Ad^®^ gauze consists of cotton gauze impregnated with paraffin.

The aim of our study was to compare these four selected gauze dressings from a technological point of view in terms of chemical and physical features, to better evaluate if and how these properties influence the process of wound healing. More in detail, our analyses were based on the evaluation of the pH and rheological behavior of the semisolid preparations that impregnate the mentioned gauzes. In addition, we performed ex vivo experiments to show the different abilities of the dressings to promote skin hydration.

## 2. Materials and Methods

### 2.1. Materials

The tested products—Betadine^®^ 10% gauze (Meda Pharma S.p.A., Milano, Italy) Connettivina^®^ Bio Plus gauze (Fidia Farmaceutici S.p.A, Albano Terme, Italy), Fitostimoline^®^ Plus gauze (Damor Farmaceutici S.p.A, Napoli, Italy), and Non-Ad^®^ gauze (Eurospital S.p.A, Trieste, Italy)—are available commercially and were kindly provided by Farmaceutici Damor S.p.A (Italy). All reagents, solvents, and other chemicals were commercial products obtained from Merck (Darmstadt, Germany). The porcine skin was provided by a local slaughterhouse (Se.ma. Sepe s.r.l, Avellino, Italy).

### 2.2. pH Measurements

For the pH determination of Connettivina^®^ Bio Plus gauze and Fitostimoline^®^ Plus gauze, 10% *w/v* solutions were prepared by scraping off the cream impregnating the gauze. For Betadine^®^ 10% gauze, the collection of the semisolid was not feasible; therefore, the gauze was immersed in 10 mL of purified water, and the resulting pH of the solution was measured. For the Non-Ad^®^ gauze, the pH was not determined since paraffin is insoluble in water.

The measurement of the pH was assessed using an LGG pH-meter 7 device (Lab Logistics Group, Meckenheim, Germany) endowed with a glass membrane electrode, preliminarily calibrated with buffers at pH = 4.00, 7.02, and 9.06.

Three different samples for each product were prepared, and for each sample, the pH measurement was performed in triplicate and the results were expressed as mean values of the triplicate tests. The final pH value was reported as mean value ± standard deviation.

### 2.3. Rheological Tests—Shear Tests

The flow tests were carried out at 25 °C and 37 °C using a Kinexus rotational rheometer (Malvern, PA, USA) equipped with a passive heat exchanger temperature-control system (KNX2500) and a parallel-plate test geometry (20 mm diameter). Samples were placed between the two plates of the rheometer, with a 1 mm gap. To prepare the semisolid formulations that impregnated the gauze, the material was extracted from the gauze itself and from the protective plastic film using a metallic spatula. Subsequently, the recovered material was mixed in a glass vial using mechanical stirring and loaded onto the rheometer plates (see GA). The flow properties of the samples were determined by assessing the trend of viscosity in response to a controlled shear rate ranging from 0.01 to 100 s^−1^. Experiments were performed in triplicate.

### 2.4. Rheological Tests—Oscillatory Tests

To assess the viscoelastic behavior of each formulation, oscillatory rheological tests were carried out through small-amplitude oscillatory shear tests in a controlled strain mode. Experiments were performed by subjecting the samples to oscillation frequencies ranging from 0.1 to 10 Hz while maintaining a constant shear strain within the linear viscoelasticity range of the material. This enabled the measurement of the elastic and the viscous moduli (G’ and G”, respectively) as a function of frequency, at 25 °C and 37 °C. As is well known, the elastic modulus represents the energy stored in the material during deformation, while the viscous modulus indicates the energy dissipated as heat. Experiments were conducted in triplicate.

### 2.5. Adherence of Semisolid Products to the Gauze

The adhesion of the semisolid products to the gauze was gravimetrically determined. Firstly, the gauze was weighed with and without the protective sheets. Then, the gauzes were cleaned with water or acetone (in the case of Non-Ad^®^ gauze) and, once dried, they were weighed. The entire procedure was repeated three times for each product to obtain an average weight of the semisolid product present on the gauze.

### 2.6. Ex Vivo Tests

#### 2.6.1. Tissue Preparation

Skin samples were kindly provided by a local slaughterhouse (Se.ma. Sepe s.r.l, Avellino, Italy). All the experiments were performed on fresh pig skin within 24 h postmortem and stored at 4 °C. Prior to experimentation, the skin was prepared by removing hair and subcutaneous fat. Pieces measuring 2 cm^2^ were cut, ensuring the inclusion of the epidermis and dermis, as previously reported [19,20]. Only the epidermis, including the stratum corneum and the dermis with a 1.7–2.3 mm thickness, were subjected to capacitance and transepidermal water-loss (TEWL) assessment for assessing skin integrity before ex vivo experimentations. Only skin explants with TEWL lower than 30 g/h∙m^2^ and corneometry higher than 50 A.U. were considered suitable for performing the skin recovery study.

#### 2.6.2. Experimental Section

Skin porcine explants were mounted on the receptor compartment of Franz vertical diffusion cells (Microglass Heim, Naples, Italy), ensuring that the upper side, the stratum corneum (SC), faced the donor compartment. Then, Connettivina^®^ Bio Plus and Fitostimoline^®^ Plus gauzes were applied on the skin surface. The receptor compartment was filled with a phosphate buffer solution with a pH of 7.4. Franz cells were placed in a thermostatic bath, at a constant temperature of 37 °C (Haake DC30; Thermo Fisher Scientific, Waltham, MA, USA), under continuous stirring on an H+P Labortechnik Variomag Telesystem (Munchen, Germany).

The collected explants (*n* = 24) were randomly divided into three groups of treatment (eight for each one):

Group C: received Connettivina^®^ Bio Plus gauze.

Group F: received Fitostimoline^®^ Plus gauzes.

Control: received no treatment.

Then, two parameters were monitored to assess the recovery action: (i) skin hydration and (ii) local skin energy balance.

Skin hydration was measured on ex vivo porcine skin explants using a Corneometer^®^ CM 825 (Courage+Khazaka Electronic GmbH, Cologne, Germany). For each measurement, the probe was applied to the skin ten times, and the average value was recorded for comparison with other checkpoints.

Analogously, skin energy was recorded by positioning a Tewameter^®^ TM Hex (Courage+Khazaka Electronic GmbH, Cologne, Germany) probe on the skin explants for 20 s after reaching the plateau. Skin readings were conducted at baseline (T0) and after 6 and 24 h of treatment (T6h and T24h) for each treatment group. Data were statistically analyzed through the Student’s *t*-test for intra-group differences vs. baseline (T0), and ANOVA was used to determine inter-group differences compared to the control.

## 3. Results and Discussion

### 3.1. pH Measurements

Fluctuations in pH significantly impact angiogenesis, collagen formation, and macrophage activity, which are crucial processes involved in wound healing. Specifically, even tiny pH changes affect the activity of matrix metalloproteinases (MMPs), which are key enzymes responsible for tissue healing and restructuring [21].

It is known that maintaining pH values close to those typically found on healthy skin (ranging from approximately 4.8 to 6) can enhance the wound-healing process. Consequently, the use of medications that create a mildly acidic environment in infected wounds can facilitate the healing process.

Conversely, an alkaline environment, which is often induced by microbial activity, is associated with chronic or difficult-to-heal wounds [5]. Indeed, an alkaline wound environment counteracts immune response, thereby promoting bacterial growth, escalating proteolytic activity, inhibiting fibroblast function, and reducing oxygen supply [22]. Moreover, bacterial growth within a wound further contributes to its alkalization, thus slowing down or compromising the healing process [23].

To assess the chemical–physical and technological features of the four selected products intended for the treatment of damaged skin, we measured the pH of aqueous solutions obtained by dissolving the semisolid preparations and soaking the gauzes. Indeed, the existing literature indicates that some dressings and topical agents may influence the wound pH, thereby promoting the healing process [22].

All the tested products yielded acidic pH values of the solutions, with values ranging from 2.66 (as determined for Betadine^®^ 10% gauze) to 4.50 (as determined for Connettivina^®^ Bio Plus gauze and Fitostimoline^®^ Plus gauze). The analysis of the obtained pH values indicates that all the formulations create an acidic milieu, which aligns with the established literature supporting the notion that such an environment contributes to the process of wound healing. This positive effect is further enhanced by the specific active ingredients found in the evaluated dosage forms and/or medical devices, each of which possesses well-established chemotherapeutic properties (see Table 2).

### 3.2. Rheology—Shear Tests

**Figure 1:**
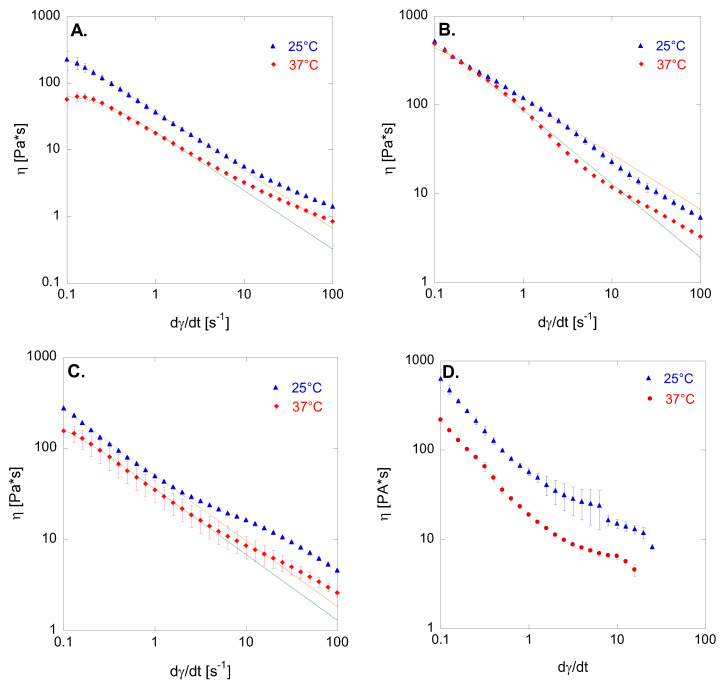
Flow curves at 25 °C and 37 °C of the cream taken from Betadine^®^ 10% gauze (**A**); Fitostimoline^®^ Plus gauze (**B**); Connettivina^®^ Bio Plus gauze (**C**); Non-Ad^®^ gauze (**D**). The results were expressed as mean values of triplicate tests ± SD. Triangles and circles represent experimental data. Solid lines are curves obtained by data fitting.

Figure 1 shows the flow curves of the creams taken from Betadine^®^ 10% gauze, Fitostimoline^®^ Plus gauze, Connettivina^®^ Bio Plus gauze, and Non-Ad^®^ gauze, at 25 °C and 37 °C.

In all cases, it was observed that the viscosity of the creams decreased with both temperature and shear rate, thereby displaying non-Newtonian, pseudoplastic behavior. This finding suggests that the analyzed samples possess favorable spreadability features, as they exhibit high viscosity at rest, facilitating adherence to the skin, while demonstrating lower viscosity under a high shear rate. Indeed, the pseudoplastic behavior can be regarded as a desirable attribute in semisolid formulations designed for cutaneous application [24].

The flow behavior of the semisolid product taken from gauzes was modelled by the Carreau equation (Equation (1)), as described previously [13].

(1) $$\frac{{\eta -\eta }_{\infty }}{\eta_{0}-\eta_{\infty }}=\dot{{\left[1+{\left(\lambda \dot{\gamma }\right)}^{2}\right]}^{\frac{m-1}{2}}}$$

where *η*_0_ is the zero-shear viscosity (Pa·s), i.e., the viscosity in the first Newtonian plateau ($\dot{\gamma }$ →0); $\eta_{\infty }$ is the viscosity (Pa·s), the viscosity in the second Newtonian plateau ($\dot{\gamma }$ →∞); *λ* is a characteristic time (s); and *m* is the flow index, or pseudoplasticity index. When *m* = 1, it describes a Newtonian beaviour, while lower *m* values are associated to an increasingly pseudoplastic rheological behaviour. Anyway, it must be underlined that in the experimental setup of this study, the second Newtonian region was not explored. Also, $\eta_{\infty }$ is negligible compared to η and $\eta_{0}$. Hence, Equation (1) can be simplified into a three-parameter model [21]:

(2) $$\eta =\dot{\eta_{0}{\left[1+{\left(\lambda \dot{\gamma }\right)}^{2}\right]}^{\frac{m-1}{2}}}$$

Additionally, the regression coefficient R^2^ was used as a measure of goodness-of-fit. The experimental data for products A, B, and C closely align with the Carreau model, as evidenced by consistently high R^2^ values. However, the viscosity of product D could not be adequately described by Equation (2), with the adjustable parameters yielding values lacking physical meaning. Despite this, all products exhibited pseudoplastic behavior, characterized by apparent shear-thinning. Table 3 summarizes the values of parameters *η*_0_, *λ*, and *m* obtained from Carreau model. Notably, *η*_0_ values were lowest for sample A, while they increased for products B and C. Additionally, the time constant *λ* serves as an indicator of the onset shear rate for shear thinning. Sample A displayed the lowest *λ* value, indicating a weaker dependence of viscosity on the shear rate compared to samples B and C. Observing the data at 37 °C, sample A demonstrated the lowest *m* value, followed by samples B and C. Thus, the optimized combination of *η*_0_ and *m* values at 37 °C is shown by sample B.

### 3.3. Rheology—Oscillatory Tests

**Figure 2:**
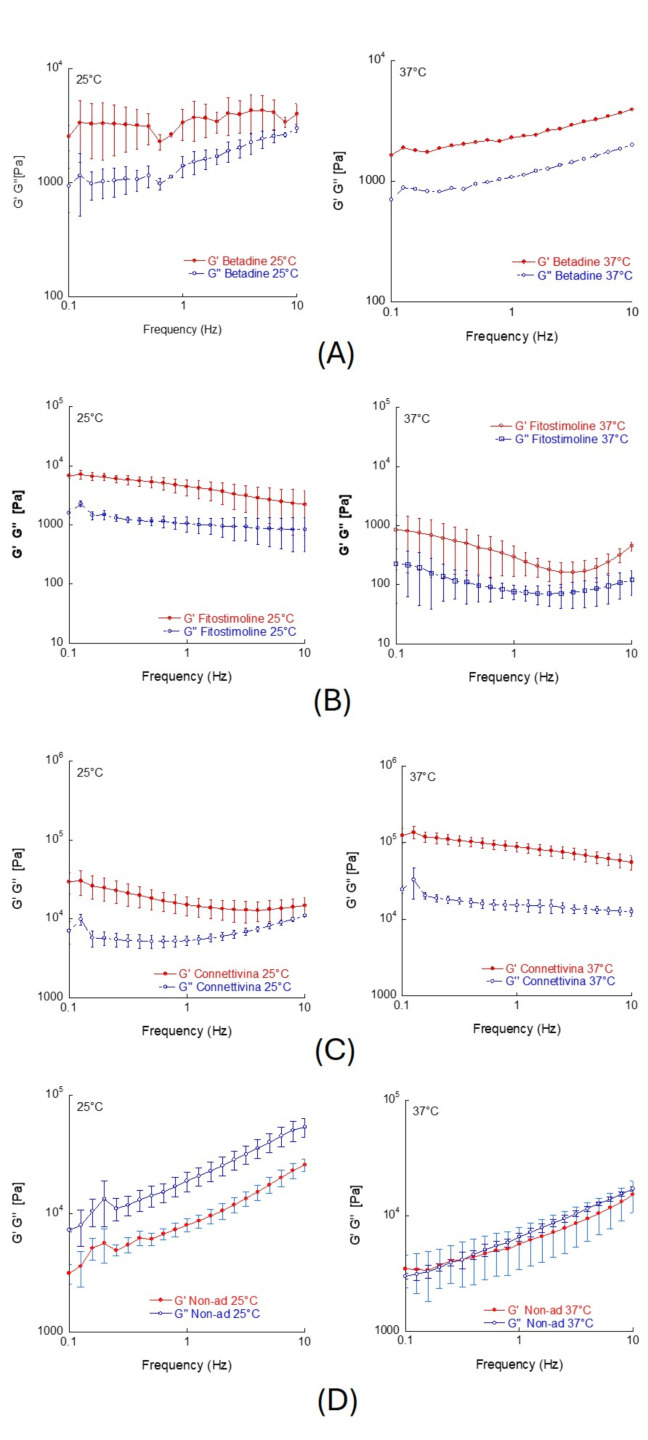
Viscoelastic moduli (G’ and G”) as a function of oscillation frequency, at 25 °C and 37 °C, of the of the cream taken from Betadine^®^ 10% gauze (**A**); Fitostimoline^®^ Plus gauze (**B**); Connettivina^®^ Bio Plus gauze (**C**); Non-Ad^®^ gauze (**D**). The values of the constant shear strain within the linear viscoelasticity regime (LVER) were 0.02 for (**A**,**B**,**D**) and 0.07 for (**C**). The results were expressed as mean values of triplicate tests ± SD.

Figure 2 presents the mechanical spectra, which depict the variation of the viscoelastic moduli (G’ and G”) with respect to the oscillation frequency for the tested creams. These spectra are shown separately for both 25 °C and 37 °C.

As shown in Figure 2D, in the case of Non-Ad^®^ gauzes, the viscous modulus (G’’) consistently surpasses the elastic modulus (G’) across the entire frequency range. Thus, the mechanical spectrum of the cream demonstrates features consistent with a viscous solution. Also, an increase in temperature leads to a reduction in the value of both the viscoelastic moduli, which maintain the same trend as the mechanical spectra and the same reciprocal ratio between the viscous and the elastic moduli (G’’ > G’) at all the analyzed frequencies.

Differently, as can be seen from Figure 2A–C, the semisolid preparations taken from the Betadine 10%, Fitostimoline^®^ Plus gauze, and Connettivina^®^ Bio Plus gauze display a rheological behavior typical of a weak gel, with G’ > G” in the entire frequency range analyzed. This rheological behavior can result from the formation of a three-dimensional macromolecular network, which arises from reversible interactions involving hydrogen bonds, van der Waals interactions, physical entanglements, hydrophobic interactions, and others. The occurrence of gel-like rheological behavior offers clear advantages for a semisolid pharmaceutical formulation intended for topical application. Indeed, such a formulation needs to create a protective barrier within the wound, also being capable of absorbing mechanical shocks and safeguarding the injured tissue [25]. Specifically, the elastic modulus provides insights into the accumulation of elastic energy during deformation.

### 3.4. Adherence of Semisolid Product to the Gauze

These tests were carried out to evaluate how much cream is lost on the protective sheets and therefore actually remains on the gauze. The results of this test are reported in Table 4.

As can be observed, the adhesion of the semisolid product to the gauze is superior in the case of Fitostimoline^®^ Plus gauze, followed by Connettivina^®^ Bio Plus gauze, Betadine^®^ 10%, and finally Non-Ad^®^ gauze. The enhanced adhesion highlighted for Fitostimoline^®^ Plus gauze may be attributed to both the characteristics of the gauze itself and to the preferable rheological properties of the semisolid product, as discussed previously. Notably, the Fitostimoline^®^ Plus gauze presents a denser weave, with a greater number of both vertical and horizontal threads, with respect to other tested products.

Thus, after considering all the obtained results, we selected the most promising products, in terms of technological characteristics, for further investigation in the study addressed to determine their efficacy on the skin.

### 3.5. Ex Vivo Experiments

**Figure 3:**
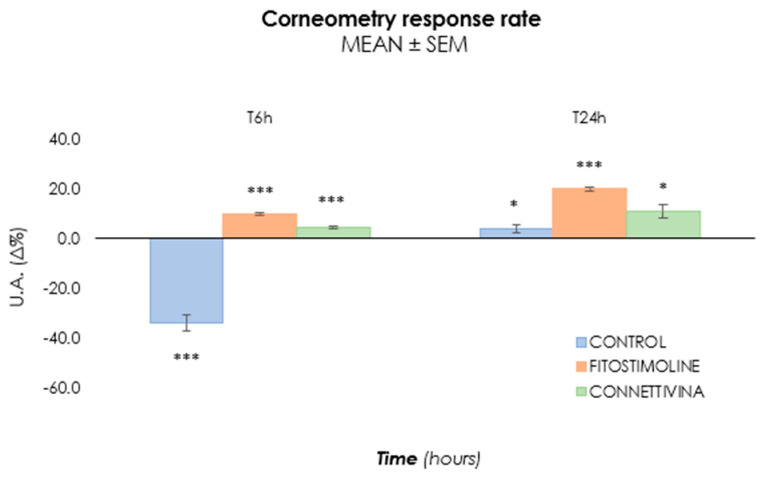
Corneometry-based assessment of ex vivo skin hydration under various treatment conditions. Data points correspond to the rate of change of corneometer measurements per hour, with error bars showing the 95% confidence interval. For the ambient condition without treatment (control), asterisks denote rates of change significantly different from zero; for all other conditions, they denote rates of change that are significantly different from the control (*: *p* < 0.05; ***: *p* < 0.001).

The effect of Connettivina^®^ Bio Plus and Fitostimoline^®^ Plus gauzes was assessed on pig-ear skin using a Franz cell apparatus. The gauze formulations were applied on the epidermis, and untreated skin was used as a control. After 6 and 24 h of contact, the gauzes were removed, and the skin samples were gently cleaned with cotton wool for the subsequent skin hydration and energy measurements. In detail, a Corneometer^®^ CM 825 assessed the hydration level of the upper skin layer, the stratum corneum (SC). This measurement is based on the capacitance property of the SC, which acts as a dielectric medium due to its water content. As is known, water has a higher dielectric constant (78.4) compared to many other substances [26,27]. The probe presents gold tracks on the top, which are separated from the skin by a glass lamina. Upon contact with the skin surface, gold tracks create an electric field between tracks with alternating attraction. The scattered field penetrates the first layer of the skin and the corneometer measures the change in the dielectric constant, which is due to skin surface hydration changing the capacitance of the precision capacitor. Skin hydration increased significantly after both Connettivina^®^ Bio Plus and Fitostimoline^®^ Plus gauze treatment compared with the baseline and control group (*p* < 0.05). Skin conductivity results, reported in Figure 3, indicate a higher water content in the stratum corneum (T6h: 10.0%; T24h: 20.0%) in the Fitostimoline^®^ Plus-treated group, which was confirmed to be two times more efficient (*p* < 0.1) than Connettivina^®^ Bio Plus in moisturizing the skin (T6h: 4.5%; T24h: 11.0%).

**Figure 4:**
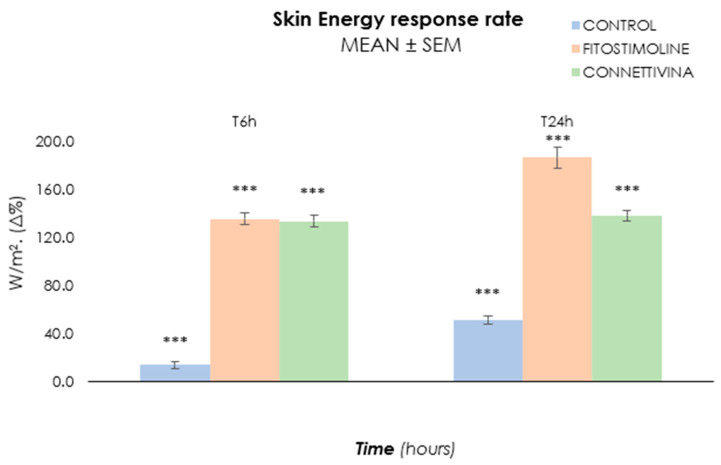
Heat loss-based assessment of ex vivo skin energy under various treatment conditions. Data points correspond to the rate of change of local energy balance measurements per hour, with error bars showing the 95% confidence interval. For the ambient condition without treatment (control), asterisks denote rates of change significantly different from zero; for all other conditions, they denote rates of change that are significantly different from the control; ***: *p* < 0.001).

Similarly, skin energy values (Figure 4) indicated that the treated group presented noticeable metabolic activity (T6h: 135.0%, T24h: 186.0% Group F; T6h: 133.0%, T24h: 138.0% Group C), thereby suggesting that both treatments were able to restore and stimulate skin vitality. Notably, Fitostimoline^®^ Plus exhibited a higher efficacy in recovering and boosting skin metabolic activity compared to Connettivina^®^ Bio Plus (+2% at T6h and +48% at T24h). In contrast, the control group maintained its metabolic activity, suggesting that the skin explant vitality was guaranteed throughout the experiment.

## 4. Conclusions

In this study, the chemical, technological, and rheological characteristics of four impregnated gauze dressings have been comprehensively examined. The results of pH measurements consistently showed an acidic environment, ranging from 2.66 to 4.50, in all the tested solutions, which is crucial for facilitating wound healing. Rheological analysis indicated pseudoplastic behavior in all semisolid preparations, with variations in the pseudoplasticity index and *η*_0_, highlighting differences in spreadability and adhesion properties. Notably, the Fitostimoline^®^ Plus gauze (sample B) exhibited a superior combination of pseudoplasticity index and *η*_0_, which lead to an enhanced adherence of the semisolid product to the gauze. In the case of the cream found on the non-paraffin gauzes, the rheological study in oscillatory mode revealed rheological behavior typical of a viscous solution, while the other products exhibited the rheological behavior of a weak gel, thereby offering advantages in creating and maintaining the wound space, as well as providing mechanical shock-absorption properties to protect the injured tissue. Ex vivo experiments showed that Fitostimoline^®^ Plus strongly enhances skin hydration and skin energy; it was proved to be two times more effective compared to Connettivina^®^ Bio Plus.

These results emphasize the importance of selecting wound dressings that not only create an optimal wound environment but also possess favorable physical properties for effective application and adherence. Overall, this study underscores the significance of considering pH regulation and rheological behavior when selecting wound dressings. These findings provide valuable insights into the optimization of wound care strategies, aiming to improve patient outcomes and enhance tissue regeneration in chronic wound management.

## Figures and Tables

**Table 1:** Composition of the tested products.

Product	Composition of Semisolid Product
Betadine^®^ 10% gauze	Povidone iodine, PEG 400, PEG 4000, PEG 6000, purified water.
Connettivina^®^ Bio Plus gauze	Hyaluronic acid sodium salt, silver sulphadiazine, PEG 4000, glycerol, purified water.
Fitostimoline^®^ Plus gauze	Rigenase^®^, polyhexanide, PEG 400, PEG 600, PEG 1500, PEG 4000, glycerin, phenoxyethanol, purified water.
Non-Ad^®^ gauze	Petrolatum, liquid paraffin.

**Table 2:** Aspect and pH determination of 10% *w*/*v* aqueous solution of semisolid preparation-soaked Fitostimoline^®^ plus gauzes and Connettivina^®^ Bio Plus gauzes. Aspect and pH determination of the solution resulting from 1 Betadine^®^ 10% gauze soaked in 10 mL of water. The final results were expressed as mean values ± SD.

Fitostimoline^®^ Plus		Connettivina^®^ Bio Plus		Betadine^®^ 10%	
Aspect (10% *w*/*v* aqueous solution)	*Opalescent*	Aspect (10% *w*/*v* aqueous solution)	*Milky*	Aspect (1 gauze soaked in 10 mL of water)	*Brown*
	pH (10% *w/v* aqueous solution)		pH (10% *w/v* aqueous solution)		pH (1 gauze soaked in 10 mL of water)
	Mean value		Mean value		Mean value
Sample 1	4.46	Sample 1	4.60	Sample 1	2.73
Sample 2	4.53	Sample 2	4.40	Sample 2	2.65
Sample 3	4.52	Sample 3	4.49	Sample 3	2.65
Mean value	4.50 ± 0.03	Mean value	4.50 ± 0.08	Mean value	2.66 ± 0.04

**Table 3:** Parameters of Carreau model for the creams taken from Betadine^®^ 10% gauze (A); Fitostimoline^®^ Plus gauze (B); Connettivina^®^ Bio Plus gauze (C); Non-Ad^®^ gauze (D).

	A		B		C		D	
	25 °C	37 °C	25 °C	37 °C	25 °C	37 °C	25 °C	37 °C
*η*_0_ [Pa∙s]	356	68.3	1238	582	9141	220	N/D	N/D
*λ* [s]	13.3	4.37	46.1	9.79	1353	12.3	N/D	N/D
m	0.126	0.120	0.382	0.170	0.280	0.279	N/D	N/D
R^2^	>0.99	>0.99	0.968	0.968	0.970	0.989	N/D	N/D

**Table 4:** Weight of the creams taken from Betadine 10% gauze, Connettivina^®^ Bio Plus gauze, Fitostimoline^®^ Plus gauze and Non-Ad^®^ gauze.

	Betadine^®^ 10% Gauze	Connettivina^®^ Bio Plus Gauze	Fitostimoline^®^ Plus Gauze	Non-Ad^®^ Gauze
Cream weight [g]	2.38 ± 0.04	2.54 ± 0.15	2.86 ± 0.05	0.96 ± 0.09
Gauze weight [g]	0.5 ± 0.01	0.7 ± 0.01	0.5 ± 0.01	0.8 ± 0.05

## Data Availability

Data are contained within the article.
